# Gender Differences in the Evaluation and Management of New Acute CHF Due to ATTRwt Cardiac Amyloidosis

**DOI:** 10.7759/cureus.59058

**Published:** 2024-04-26

**Authors:** Michael Fragner, Jude Elsaygh, Sudarshan S Srivats, Kevin Pink

**Affiliations:** 1 Internal Medicine, New York-Presbyterian Brooklyn Methodist, Brooklyn, USA; 2 Internal Medicine, Catholic Medical Center, Manchester, USA

**Keywords:** gender differences, adult cardiac disease, amyloid, cardiac amyloid, transthyretin amyloid cardiomyopathy

## Abstract

Cardiac amyloidosis can be grouped into two main categories: immunoglobulin light chain (AL) and transthyretin (hATTR or hereditary and ATTRwt or wild type). Cardiac infiltration of misfolded proteins can lead to significant infiltrative processes and subsequent heart failure. Diagnosis of ATTRwt heavily relies on clinical suspicion, as it typically appears later in life and is limited to the heart. It is routinely reported that ATTRwt significantly affects males more than females; however, older patients diagnosed with ATTRwt and those diagnosed at autopsy are significantly more likely to be female. Earlier, a more precise diagnosis in females could detect disease at an earlier stage and expedite treatment.

## Introduction

There is a discrepancy between males versus females in the diagnosis of ATTRwt regarding the onset of diagnosis, posthumous diagnosis, and more advanced stage of disease diagnosis, with females leading in all categories. This leaves questions to be asked: why are we not diagnosing females earlier, and would earlier diagnosis lead to improved clinical outcomes and length of survival?

It has been found that older patients diagnosed with ATTRwt are significantly more likely to be female, and patients diagnosed with ATTRwt at autopsy were also more likely to be female [1]. In addition to being older at the time of diagnosis, women have been shown to have more advanced disease in ATTRwt at the time of diagnosis with greater concentric hypertrophy, higher LV filling pressures, thicker interventricular septum, worse diastolic dysfunction (higher grade), and worse RV systolic function on transthoracic echocardiogram (TTE) [2]. Awareness of red flags in women, especially those older than 60-70 years old, with unexplained heart failure, could be a key change to how we reduce the underdiagnosis of ATTRwt [3]. Keeping in mind both cardiac manifestations and extracardiac (carpal tunnel syndrome, spinal canal stenosis, and tendinopathies with spontaneous rupture, especially the biceps tendon) is an important screening point that can easily be overlooked [4].

The diagnosis of ATTRwt has historically been dominated by males (between 25 and 50:1 male-to-female ratio) [5]. In a meta-analysis estimating the gender distribution of patients with ATTRwt, Kroi et al. found a male proportion of diagnoses in ATTRwt to be 86.9%, with a 7:1 male-to-female ratio [1]. This imbalance could be attributed to biological sex hormones, with estrogen potentially serving as a cardioprotective component [6]. However, a singular hormonal component has not been conclusively determined, and this diagnostic disparity in ATTRwt is more likely impacted by extrinsic factors and diagnostic shortcomings [6]. The age of diagnosis could be attributed to survival bias as women, on average, tend to live longer than men. However, after controlling for age, Kroi et al. still were able to show that women were significantly diagnosed more at autopsy than men [1].

## Case presentation

**Figure 1:**
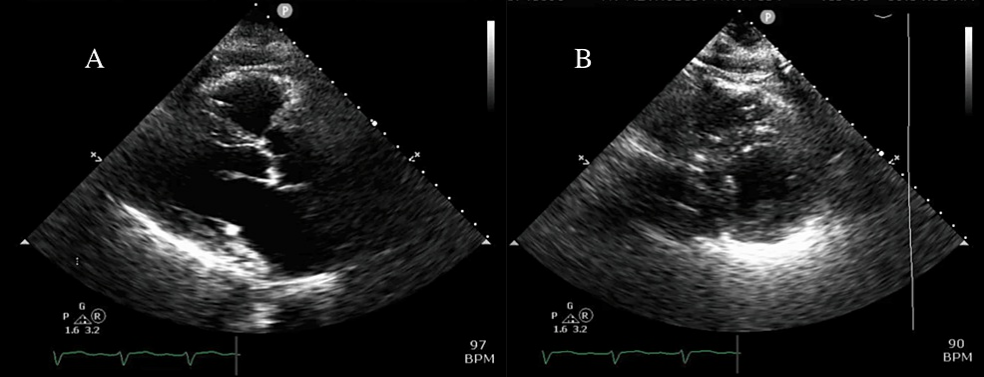
Transthoracic echocardiogram Parasternal long axis view (A) and parasternal short axis view (B) demonstrating moderate concentric left ventricular hypertrophy, a moderately dilated left ventricle, and an interventricular septal diameter of 1.2 cm.

A 79-year-old female with a history of heart failure with reduced ejection fraction (HFrEF) of 20-25% due to non-ischemic cardiomyopathy of unknown origin, hypertension, diabetes, and asthma presented with non-radiating, non-exertional, dull substernal chest pain, and dyspnea on exertion. Physical exam was notable for end-expiratory wheeze and lower extremity edema. A chest X-ray showed mild pulmonary congestion and cardiomegaly. EKG showed normal sinus rhythm, left ventricular hypertrophy (LVH), left axis deviation, left anterior fascicular block, V2 and V3 1.5 mm ST elevations, and septal Q waves in V1 and V2 (pseudo-infarct pattern). B‐type natriuretic peptide was unremarkable, and troponins were not elevated. TTE demonstrated an improved ejection fraction of 35%, moderate global hypokinesis of the left ventricle, moderate concentric LVH, moderately dilated left ventricle, and an interventricular septal diameter of 1.2 cm (Figure 1). She was concomitantly in asthma exacerbation, which resolved after a course of nebulizers and steroids. 

**Figure 2:**
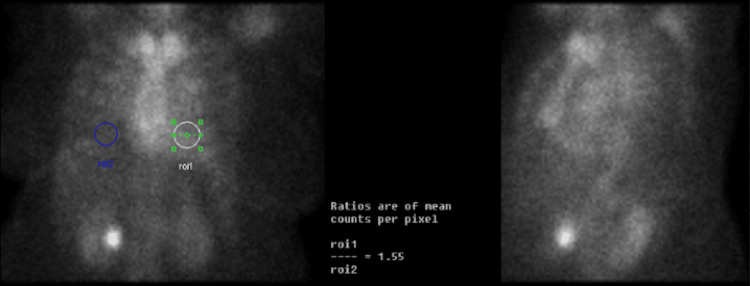
Technetium-99m pyrophosphate scan The heart-to-contralateral lung (H/CL) ratio using region of interest (ROI) was 1.55, indicating focal myocardial uptake of technetium-99m pyrophosphate.

**Figure 3:**
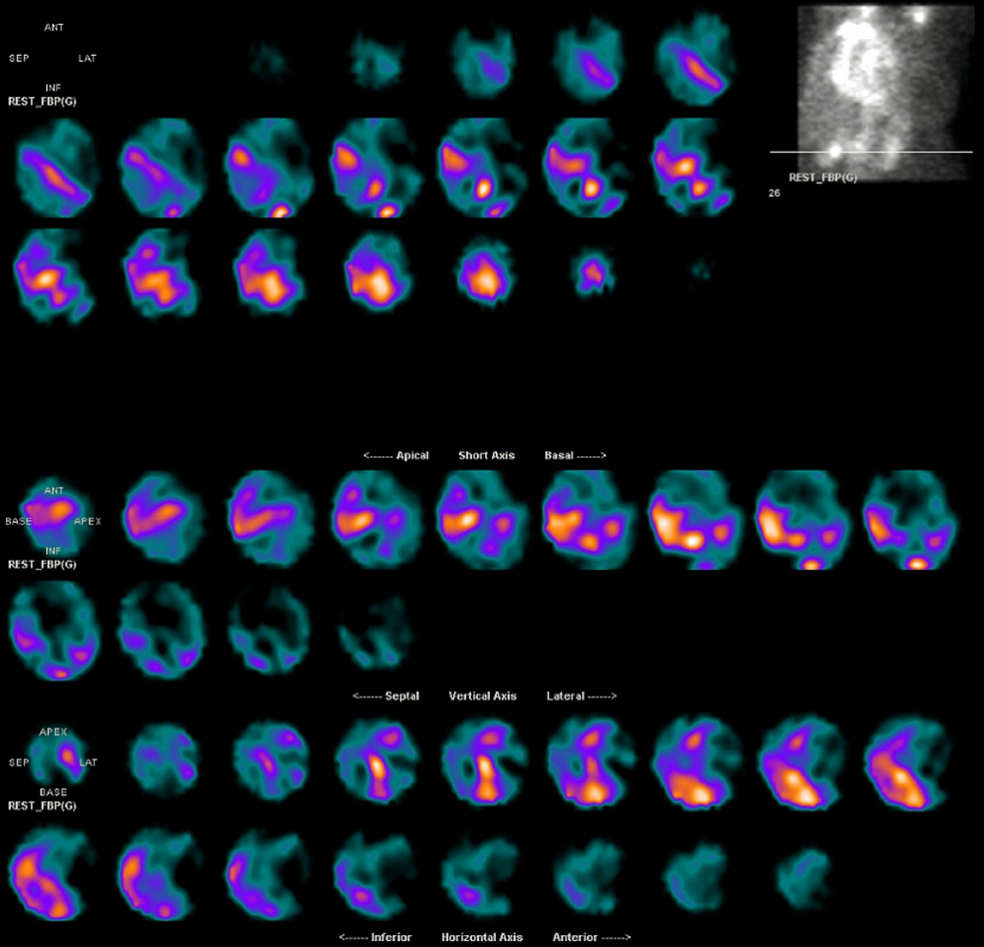
Single-photon emission computed tomography (SPECT) imaging of the myocardium Three-dimensional display confirming uptake of radioactive tracer in the myocardium.

The patient was unable to tolerate lying flat for a nuclear medicine (NM) stress test for ischemic evaluation due to orthopnea. She underwent a coronary angiogram revealing non-obstructive disease and post-capillary WHO group 2 pulmonary hypertension (pulmonary artery mean pressure of 27, pulmonary capillary wedge pressure of 20, and pulmonary vascular resistance of 1.2) with elevated right-sided and left-sided filling pressures (15 mmHg in right atrium, 37/14 mmHg in right ventricle, and pulmonary capillary wedge pressure of 20 mmHg). Because of her unremarkable ischemic evaluation, unrevealing non-ischemic cardiomyopathy workup, concentric LVH, and borderline-elevated interventricular septum diameter thickness of 1.2 cm as seen on TTE, she was referred for a technetium-99m pyrophosphate (PYP) scan to evaluate for amyloidosis. Subsequent PYP imaging strongly suggested ATTR amyloidosis (Figure 2) with a heart-to-contralateral lung (H/CL) ratio of 1.55, and subsequent single-photon emission computed tomography (SPECT) confirmed radioactive tracer uptake in the myocardium (Figure 3). Both amyloid light chain (AL) amyloidosis and hATTR were ruled out with a negative myeloma workup (serum and urine immunofixation and serum-free light chain assay) and genetic testing, respectively. She was continued on sacubitril-valsartan, furosemide, and spironolactone for volume management as tolerated and was to have close follow-up with the advanced heart failure service for initiation of tafamidis and symptom management. Unfortunately, the patient did not continue following up with her cardiologists after persistent efforts from her providers and was never started on tafamidis.

## Discussion

The differences in ATTR-cardiomyopathy (CM) between men and women have been minimally explored; however, there is growing evidence of sex-based structural and functional differences. From an objective standpoint, women are more likely to have a lower interventricular septal and posterior wall thickness, lower left ventricular end-diastolic diameter, and higher left ventricular ejection fraction in both normal and diseased hearts [7]. LV wall thickness >12 mm is a general threshold for evidence of ATTR-CM, and given the tendency for females to have smaller cardiac anatomy at baseline, they are at risk for echocardiographic underdiagnosis [8]. Although all-cause mortality has not been shown to be significantly affected, more advanced disease on echocardiography and higher NAC scores (National Amyloidosis Center scores) have been seen in women compared to men [2].

We must keep in mind that there may be additional cardioprotective factors that delay the progression of the disease, such as the genomic and non-genomic effects of estrogen. Estrogen’s presumed protective role in cardiovascular disease in premenopausal patients is based on its ability to increase angiogenesis and vasodilation and decrease reactive oxygen species, oxidative stress, and fibrosis [9,10]. This can be supported by studies showing a higher proportion of females diagnosed with ATTRwt and advanced cardiomyopathy at post-menopausal age with a less severe functional and structural echocardiographic profile [10].

This places women at a distinct disadvantage, as their echocardiogram findings may not be impressive enough to warrant further investigation. By taking a more aggressive approach with a female patient with heart failure symptoms without a known etiology (ischemia ruled out and unremarkable non-ischemic workup) and borderline early imaging findings (i.e., LV thickness within 2-3 mm of 12 mm threshold, concentric thickening of the LV, a degree of diastolic dysfunction, RV hypertrophy, bi-atrial enlargement, restrictive filling pattern on TTE, apical sparing pattern on TTE, and pseudo-infarct ECG pattern), diagnosing may become more effective, inclusive, and prompt [11]. 

It would be prudent to make changes to diagnostic criteria to help reduce underdiagnosis when using non-invasive methods. This could be done by altering the cut-off values or cardiac dimensions by sex and body surface area when utilizing echocardiography, more frequent use of NM imaging (especially in ruled-out AL amyloidosis), and improved clinical awareness in women with unexplained heart failure and borderline evidence [8]. Having a lower threshold for patients to undergo non-invasive testing may be the key to changing the theme of female underdiagnosis. In one study analyzing patients with suspected and confirmed ATTR (via endomyocardial biopsy), there was 100% specificity in diagnosing ATTRwt on NM imaging when meeting the criteria of HF on echocardiogram or cardiac MRI suggestive of amyloidosis and absence of monoclonal antibodies ruling out AL amyloidosis [12].

Some studies have suggested a difference in the manifestation of symptoms based on gender, with female patients having a more atypical presentation [13]. Takashio et al. were able to show that ATTRwt-diagnosed females had higher BNP levels, moderate to severe aortic stenosis more frequently, and smaller LV size and thickness. Additionally, not one female had LVH as a manifestation that led to a diagnosis [13]. 

Cardiac amyloidosis heavily relies on a clinician’s judgment. González-López et al. showed that up to 35% of cases had been misdiagnosed with other cardiovascular diseases (i.e., hypertensive cardiomyopathy, hypertrophic cardiomyopathy, ischemic heart disease, and aortic stenosis) [14]. Having a low threshold for suspicion, given red flags in the setting of borderline suspicious TTE findings, is vital for improved diagnosing. Such red flags could include heart failure with preserved ejection fraction (HFpEF) in the absence of hypertension, bi-atrial enlargement, intolerance of CHF medications, arrhythmias (especially atrial fibrillation and conduction disease), hypotension in previously hypertensive patients, pseudo-infarct on EKG, and reduced longitudinal strain with apical sparing on strain imaging [3,4,15]. 

In amyloidosis, patients already have smaller ventricles with poor stroke volume. Since there is a reliance on heart rate for cardiac output, decreasing the heart rate with medication such as beta blockers can worsen symptoms [15]. Given amyloidosis’ effect on the autonomic system and the hypotensive effects of sacubitril-valsartan (angiotensin receptor-neprilysin inhibitors or ARNIs), its benefit in HFpEF has yet to be elucidated [15]. This was likely the confounding aspect in the PARAGON trial, which concluded an ineffectiveness of sacubitril-valsartan in decreasing admissions due to heart failure or death from cardiovascular etiology in HFpEF [16]. This has largely been attributed to the idea of a large subset of patients in this trial with underlying, undiagnosed cardiac amyloidosis. This is due to the similarities in presentation, given that both diseases can manifest with increased septal thickness, left atrial enlargement, and diastolic dysfunction [16].

The American College of Cardiology writing committee’s expert consensus decision pathway cautions against the use of guideline-directed therapy for cardiac amyloidosis [17]. For reasons as stated above, there is a consensus agreement that beta-blockers should be used with caution and are likely to be poorly tolerated, and discontinuation likely improves outcomes. ARNIs, angiotensin-converting enzyme (ACE) inhibitors, and angiotensin receptor blockers (ARBs) have also been shown to be poorly tolerated due to their vasodilatory effects [17]. Interestingly, mineralocorticoid antagonists showed a reduction in cardiovascular death, CHF hospitalization, or aborted cardiac arrest as seen from results of the TOPCAT trial, a retrospective analysis of HFpEF patients with a large population of echocardiographic determined (but not confirmed) cardiac amyloidosis patients [17,18]. In addition, the writing committee concluded that there was insufficient evidence regarding the efficacy or harm of sodium-glucose cotransporter inhibitors in cardiac amyloidosis [17].

There is also a demonstrated high rate of arrhythmias in cardiac amyloidosis, often symptomatic and difficult to treat. Conduction disease and ventricular arrhythmias are common; however, there is limited data on the usage of internal cardioverter-defibrillators or pacemakers and their effect on mortality [19]. Atrial tachyarrhythmias impose great difficulty in terms of treatment as well, given the restrictive pathophysiology of CA and reliance on heart rate. There is a general consensus of a preference for rhythm control due to cardiac output reliance on heart rate and that patients may be better served in sinus rhythm. With new strategies and pharmacology working to improve mortality in CA patients, a historically underdiagnosed and understudied population, further studies are needed to improve guidelines on the management of arrhythmias in CA patients [19].

In a retrospective study of 160 patients at a tertiary unit, time to diagnosis of ATTRwt from symptom onset was positively correlated with all-cause mortality. For each month after symptom onset, each patient’s risk of death increased by 5%. Getting diagnosed greater than six months from symptom onset had a median survival rate of 2.5 years, and patients who were diagnosed earlier were associated with a greater median survival rate [15]. Being diagnosed earlier allows for better control of progression with disease-modifying drugs such as tafamidis, an FDA-approved amyloid stabilizer shown to reduce mortality and cardiovascular disease-related hospitalizations, in addition to less time spent inappropriately treating conditions that are often being misdiagnosed [15,20].

## Conclusions

There undoubtedly may be underlying biases when approaching amyloidosis. Do clinicians approach patients with cardiac disease the same, and do primary non-cardiology-specific clinics have the same low threshold for further investigation? With improving non-invasive diagnostic modalities and disease-modifying treatments, having a low threshold for ATTR consideration in the female population (improved clinical suspicion and imaging criteria) could help rectify a diagnostic imbalance in women. By identifying patients with advanced disease, we can closely study methods to more appropriately diagnose future patients and diagnose them at an earlier, less severe stage.
